# The CbbQO-type rubisco activases encoded in carboxysome gene clusters can activate carboxysomal form IA rubiscos

**DOI:** 10.1016/j.jbc.2021.101476

**Published:** 2021-12-08

**Authors:** Yi-Chin Candace Tsai, Lynette Liew, Zhijun Guo, Di Liu, Oliver Mueller-Cajar

**Affiliations:** School of Biological Sciences, Nanyang Technological University, Singapore, Singapore

**Keywords:** rubisco, rubisco activase, AAA+ ATPase, carboxysomes, carbon fixation, CABP, carboxy-arabinitol 1,5-bisphosphate, CCM, carbon dioxide concentrating mechanism, ECM, Enzyme-CO_2_-Mg^2+^, ECMC, ECM-CABP, ER, enzyme–RuBP, MOE, Ministry of Education, NRF, National Research Foundation, Rca, rubisco activase, RuBP, ribulose 1,5-bisphosphate

## Abstract

The CO_2_-fixing enzyme rubisco is responsible for almost all carbon fixation. This process frequently requires rubisco activase (Rca) machinery, which couples ATP hydrolysis to the removal of inhibitory sugar phosphates, including the rubisco substrate ribulose 1,5-bisphosphate (RuBP). Rubisco is sometimes compartmentalized in carboxysomes, bacterial microcompartments that enable a carbon dioxide concentrating mechanism (CCM). Characterized carboxysomal rubiscos, however, are not prone to inhibition, and often no activase machinery is associated with these enzymes. Here, we characterize two carboxysomal rubiscos of the form IA^C^ clade that are associated with CbbQO-type Rcas. These enzymes release RuBP at a much lower rate than the canonical carboxysomal rubisco from *Synechococcus* PCC6301. We found that CbbQO-type Rcas encoded in carboxysome gene clusters can remove RuBP and the tight-binding transition state analog carboxy-arabinitol 1,5-bisphosphate from cognate rubiscos. The *Acidithiobacillus ferrooxidans* genome encodes two form IA rubiscos associated with two sets of *cbbQ* and *cbbO* genes. We show that the two CbbQO activase systems display specificity for the rubisco enzyme encoded in the same gene cluster, and this property can be switched by substituting the C-terminal three residues of the large subunit. Our findings indicate that the kinetic and inhibitory properties of proteobacterial form IA rubiscos are diverse and predict that Rcas may be necessary for some α-carboxysomal CCMs. These findings will have implications for efforts aiming to introduce biophysical CCMs into plants and other hosts for improvement of carbon fixation of crops.

The CO_2_-fixing enzyme rubisco catalyzes the most quantitatively significant conversion of CO_2_ gas to biomass, operating in the majority of autotrophic organisms. All genuine rubiscos possess an ancient and conserved active site architecture and a complex reaction mechanism (1). An inevitable consequence is a side reaction with oxygen (2), leading to the production of the metabolite 2-phosphoglycolate, which must be recycled *via* salvage pathways (“photorespiration”) to avoid the loss of carbon from the Calvin cycle (3). It is thought that the emergence of rubisco prior to the oxygenation of earth's atmosphere predisposed the enzyme to display a limited capacity to adapt to modern atmospheric conditions (4, 5). To compensate, diverse clades of rubisco-dependent autotrophs readily and convergently evolved a broad suite of carbon dioxide concentrating mechanisms (CCMs), many of which rely on spatial compartmentation of the carboxylase (6). A solution encountered in all cyanobacteria and many proteobacteria involves sequestration of cellular rubisco in protein-encapsulated microcompartments known as carboxysomes (7, 8, 9).

Rubisco requires the cofactors CO_2_ and Mg^2+^ to bind at the active site prior to becoming catalytically competent (10). It has recently emerged that most if not all rubiscos are prone to binding of the substrate ribulose 1,5-bisphosphate (RuBP) to the apoenzyme, leading to formation of inhibited complexes of various stability (11, 12). Inhibited rubisco complexes can also form when a suite of other phosphorylated sugars binds to either the apoenzyme or holoenzyme. These include the rubisco “misfire” products xylulose 1,5-bisphosphate (13) and d-glycero-2,3-pentodiulose-1,5-bisphosphate (14) or the night-time inhibitor in plants: 2-carboxy-d-arabinitol 1-phosphate (15). Inhibited rubisco complexes are recognized and remodeled by a growing suite of diverse molecular chaperones, known as the rubisco activases (Rcas). All the characterized Rca proteins belong to the superfamily of AAA+ ATPases (16, 17) but have been co-opted to the Rca function at least three times independently (11). Rca function involves ATP hydrolysis, which is coupled to rubisco active site rearrangements that release the inhibitory sugars (18).

Proteobacterial genomes frequently encode up to three *bona fide* rubisco enzymes (19, 20, 21, 22), which in some cases have been shown to be differentially expressed according to factors such as the environmental CO_2_ concentration (23, 24, 25). Form I rubiscos encoded in α-carboxysomal gene clusters are referred to as form IA^C^, whereas those not associated with carboxysome genes are form IA^Q^ rubiscos (19). *Acidithiobacillus ferrooxidans* possesses three operons encoding three different rubiscos (25). Each cluster also contains single copies of distinct *cbbQ* and *cbbO* genes. We have previously characterized the gene products of *cbbQ* and *cbbO* found in two clusters (encoding form IA^Q^ and form II rubiscos) and demonstrated biochemically that CbbQO complexes functionally form a third class of Rca (26). Mechanistically, CbbQO-type Rcas are highly distinct to the other two classes; they belong to the MoxR family of AAA+ proteins (27, 28) and rely on the CbbO adaptor protein for function. CbbO binds rubisco *via* its von Willebrand factor A domain, and a single CbbO subunit binds to the conserved concave surface of the CbbQ hexamer (29).

The *cbbQ* and *cbbO* genes found in the *A. ferrooxidans* carboxysomal cluster (Fig. 1) are therefore likely to encode an Rca that specifically remodels inhibited carboxysomal form IA^C^ rubiscos. This hypothesis was tested by Sutter *et al.* for *Halothiobacillus neapolitanus*; however, the reported activase assays used purified CbbQ complexes lacking the essential CbbO subunit. Analysis of fractured carboxysomes in that work showed that CbbQ associated with a shell enriched pellet fraction, suggesting an interaction of CbbQ with the carboxysome shell (30, 31).

**Figure 1:**
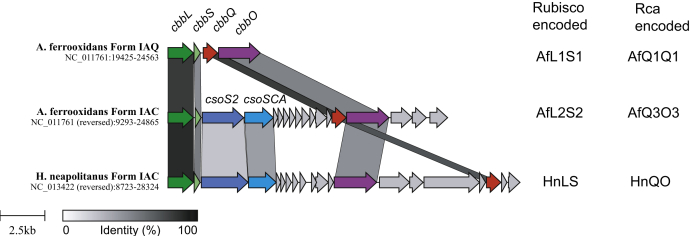
**Form I rubisco-associated operons in *Acidithiobacillus ferrooxidans* and *Halothiobacillus neapolitanus*.** The relative position and sequence identity between rubisco (*cbbL* and *cbbS*) and activase (*cbbq* and *cbbO*) encoding genes is indicated. The terminology used in this work for the different rubiscos and Rca complexes encoded in the operons is also shown. Rca, rubisco activase.

A caveat regarding the need for carboxysomal Rcas relates to the fact that previously examined carboxylases (belonging to the form IB clade) do not form stably inhibited complexes with the substrate RuBP (32, 33, 34). In addition, the frequent absence of candidate Rca genes in the genomes of cyanobacteria and proteobacteria that utilize carboxysomes suggest that Rca function is not strictly required for the carboxysomal CCM (35, 36). The existence of carboxysomal Rca systems will require ATP and ADP to pass the carboxysomal shell, which is widely believed to be selectively permeable and possibly even gated (37). With regard to form IB rubisco, it has very recently been shown that the Rca-like protein found in some cyanobacterial clades (38, 39) is a functional Rca in *Nostoc* sp. PCC7120 (40). Its mechanism involves an interaction with the rubisco large subunit N terminus, which is conserved in the higher plant system (41). The homolog encoded by *Fremyella diplosiphon* showed the expected carboxysomal localization (35).

Recently, there have been tremendous advances toward introducing carboxysomes and their associated rubiscos into higher plants (42, 43). In conjunction with a functional CCM, it is predicted that such a modification could enhance photosynthetic carbon assimilation of a C3 plant by 60% (44, 45). It has even been possible to transfer a powered and functional carboxysome CCM into *Escherichia coli* (46). In order to fully capitalize on these advances, it is imperative to resolve and define the role of Rcas in such compartments.

Here, using pure components, we functionally characterize the properties of rubiscos and define the Rca function of CbbQO complexes encoded in the carboxysomal clusters of *A. ferrooxidans* and *H. neapolitanus*, respectively. We found that both chaperones are indeed capable of removing both loose-binding RuBP and the exceptionally tight-binding transition-state analog carboxy-arabinitol 1,5-bisphosphate (CABP) from form IA^C^ rubisco active sites. The specificity of the two CbbQO complexes associated with the two form IA rubisco operons in *A. ferrooxidans* can be switched *via* amino acid substitutions in the RbcL C terminus. We propose that carboxysomal rubiscos exist on a continuum related to their kinetic properties and tendencies to bind inhibitors. Their position on the continuum determines the extent of activase dependency and permits activase loss in organisms possessing high-velocity and low-efficiency carboxylases.

## Results

### Bioinformatics analysis of form IA rubiscos and associated CbbQO sequences

We performed BLAST homology searches using the *A. ferrooxidans* IA^Q^ large subunit sequence (UniProt: B7JA24) to collect form IA CbbL sequences. The sequences were then classified according to their gene neighborhoods. Form IA^C^ sequences were followed by *csoS2* and other carboxysome genes and contained *cbbQ* and *cbbO* in the operon. Form IA^Q^ genes were directly adjacent to *cbbQ* and did not encode carboxysome genes (Fig. 1). Among α-carboxysome clusters encoding form IA^C^ rubisco and CbbQ and CbbO, there appear to be two arrangements. In one cluster type, *cbbQ* and *cbbO* genes are positioned directly downstream of the shell-encoding genes. In the other cluster type, *cbbO* and *cbbQ* are separated by genes encoding the dab complex (Fig. 1) (47).

We decided to introduce a new form IA^C^ division: these constituted form IA CbbL sequences encoded in carboxysomal clusters that did not contain *cbbQ* and *cbbO* genes and were designated as IAC_Q^−^. Form IA^C^ and IA^Q^ sequences are all proteobacterial, whereas the IAC_Q^−^ sequences are also found in α-cyanobacteria and the endosymbiont of *Paulinella chromatophora* (48). A maximum likelihood tree indicated that form IA^C^ and form IA^Q^ CbbL sequences do not form distinct clades, as observed previously (19) (Fig. S1*A*). In contrast, form IAC_Q^−^ large subunits formed a well-supported separate clade. In addition, the multiple sequence alignment (Fig. S2) revealed that all the form IA^C^ and IA^Q^ CbbL sequences possessed a C-terminal motif (KLDXXHK or KLDXXXR) consistent with the experimentally characterized CbbQO interaction (26). In contrast, all examined form IAC^Q−^ sequences had diverse C termini, which generally terminated two residues earlier (KLDXX) (Fig. S2).

The phylogenetic relationship between form IA rubisco-associated CbbS (Fig. S1*B*), CbbQ (Fig. S1*C*), and CbbO (Fig. S1*D*) amino acid sequences could not be used to predict whether they were associated with carboxysomal clusters (IA^C^ or IA^Q^ rubiscos). There is therefore no support for distinct ancestral form IA^Q^ or IA^C^ rubisco/activase systems. Instead form IA-associated CbbQO activase components appear to change their context over time, which would lead one to expect biochemical compatibility between non-native rubisco–Rca pairs.

### The inhibition and reaction kinetics of CbbQO-associated form IA rubiscos are diverse

**Figure 2:**
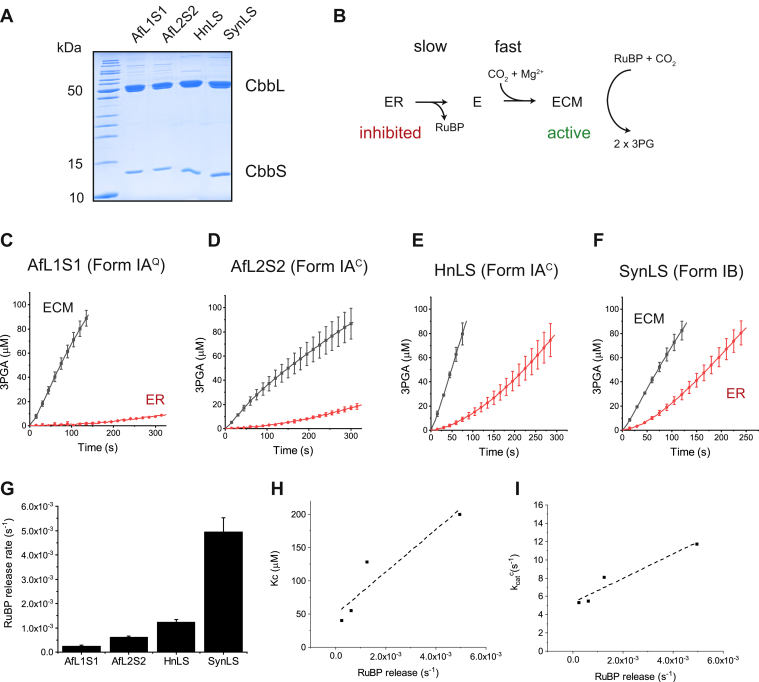
**CbbQO-associated form IA rubiscos possess diverse inhibitory properties.***A*, SDS-PAGE analysis of purified rubiscos. *B*, scheme describing the relationship between inactive enzyme–RuBP (ER) complex and the active (ECM) holoenzyme. *C*–*F*, inhibitor release kinetics of different form I enzymes is variable. Carboxylation time courses of 0.1 μM of fully activated holoenzyme (ECM) or apo-rubisco–RuBP (ER) complex are shown (50 mM NaHCO_3_ and 1 mM RuBP). Error bars signify mean and standard deviation of three technical replicates. *G*, the RuBP release rate was derived from the ER time courses shown in *C*–*F*. *H* and *I*, scatterplot of RuBP release rate against *K*_*m*_ (CO_2_) (*H*) and carboxylation rate (*I*) for the four enzymes. ECM, Enzyme-CO_2_-Mg^2+^; RuBP, ribulose 1,5-bisphosphate.

Existing data suggest that carboxysomal form I rubiscos do not form tight-binding complexes with RuBP in the apo state but readily release the inhibitor. This property correlates with high carboxylation velocities and Michaelis constants for CO_2_, but this generalization is exclusively derived from cyanobacterial form IB enzymes (32, 33, 40, 49, 50). It is not known whether carboxysomal form IA^C^ rubiscos follow the same trend. We therefore decided to compare kinetic parameters and the spontaneous release of RuBP for the different form IA enzymes encoded in *A. ferrooxidans*, the carboxysomal form IA^C^ rubisco from *H. neapolitanus* and the carboxysomal form IB model enzyme from *Synechococcus* PCC6301. Following purification (Fig. 2*A*), the apoenzymes were preincubated with RuBP to form enzyme–RuBP (ER) (Fig. 2*B*) and subsequently added to a rubisco assay mixture. The activity of the ER complex (*red traces*) was then compared with that of the preactivated Enzyme-CO_2_-Mg^2+^ (ECM) holoenzyme (*black traces*) allowing the rate of RuBP release to be estimated by quantifying the formation of active ECM holoenzymes (32, 51) (Fig. 2*B*).

As reported earlier (26, 52, 53), the form IA^Q^ rubisco (AfL1S1) formed a stable binary complex with RuBP and had the slowest inhibitor release rate (Fig. 2*C*). The three carboxysomal enzymes displayed faster but highly variable reactivation rates (Fig. 2, *D*–*G*). The activity of fully activated AfL2S2 form IA^C^ enzyme was not linear over the time course, indicating a loss of functional active sites over the reaction time course (the “fallover” phenomenon commonly observed in higher plant rubiscos (32, 51)). Compared with the very loose RuBP-binding phenotype of the cyanobacterial form IB SynLS rubisco, all form IA enzymes assayed here had much slower RuBP release rates and would therefore be predicted to benefit from an associated activase system.

Kinetic characterization of the form IA enzymes indicated that the two *A. ferrooxidans* rubiscos had highly similar carboxylation velocities (∼5.5 s^−1^) and Michaelis constants (40–50 μM) for CO_2_ (Table 1 and Fig. S3). The carboxysomal AfL2S2 form IA^C^ enzyme had a much reduced specificity factor of 32, compared with 56 for the form IA^Q^ rubisco. *H. neapolitanus* form IA^C^ (HnLS) rubisco kinetics were distinct, being faster and possessing a much higher Michaelis constant for CO_2_. Compared with the fast model carboxysomal form IB Synechococcus enzyme SynLS (54, 55, 56), HnLS displayed an intermediate phenotype (Table 1).

**Table 1:** Kinetic parameters of the form I rubiscos

Rubisco	$k_{\text{cat}}^{\text{c}}$ (s^−1^)	*K*_*C*_ (μM)	$k_{\text{cat}}^{\text{c}}/K_{\text{C}}$ (s^−1^.mM^−1^)	*S*_C/O_
AfL1S1	5.2 ± 0.1	40 ± 3	130	56.4 ± 1.7
AfL2S2	5.5 ± 0.1	55 ± 3	100	32.2 ± 0.5
HnLS	8.0 ± 0.4	127 ± 16	63	43.6 ± 1.1
SynLS^a^	11.7 ± 0.6	200 ± 4	59	42.7 ± 2.8

Michaelis–Menten plots used to derive the parameters are shown in Fig. S3.

a Data from the study by Davidi *et al.* (56).

The observed general trend of increased carboxylation velocities and reduced specificity factors for the carboxysomal enzymes is commonly observed for rubiscos that are supplied by CCMs. However, the three carboxysomal enzymes characterized here possessed a significant diversity in their kinetics. For the four enzymes evaluated here, there was a clear correlation between RuBP release rate and both the *K*_*m*_ (CO_2_) and carboxylation turnover rate (Fig. 2, *H* and *I*). This observation is consistent with the hypothesis by Tcherkez *et al.* (57), which predicts that inhibitor binding should be enhanced in rubiscos that display high affinities for CO_2_ and the associated low carboxylation velocities.

The small selection of kinetic data and inhibitory properties shown here and reported in the literature (42, 52, 53, 58, 59, 60, 61) suggest that the form IA clade of rubiscos are likely to have diverse kinetics related to the relative ability of host organisms to acquire and concentrate CO_2_.

### The CbbQO complexes encoded in carboxysome clusters are functional Rcas

**Figure 3:**
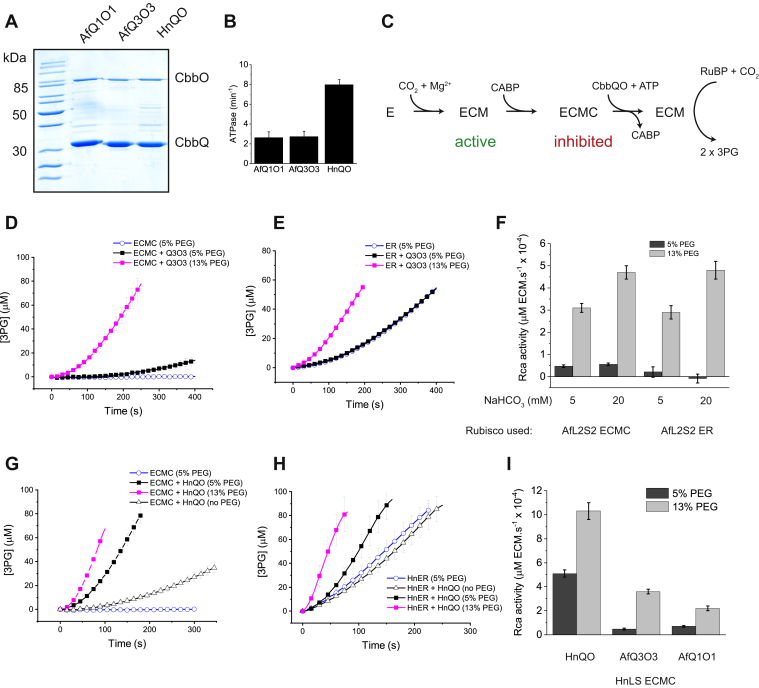
**Carboxysomal CbbQO-type rubisco activases are functional.***A*, SDS-PAGE analysis of purified CbbQO activases. *B*, ATPase activity of the CbbQO complexes expressed per protomer CbbQ. *C*, experimental scheme of the rubisco activase assays. Apo-rubisco (E) is activated using the nonsubstrate CO_2_ and Mg^2+^ cofactors to form ECM. The transition-state analog CABP stoichiometrically binds to ECM to form inactive ECMC. Addition of a compatible CbbQO activase and ATP releases CABP to recover ECM, which produces 3PG in the spectrophotometric assay. Inhibited carboxysomal rubiscos (0.3 μM ECMC [*D*] or ER [*E*] active sites) from *Acidithiobacillus ferrooxidans* were assayed at 20 mM NaHCO_3_ in the absence and presence of the cognate CbbQO activase AfQ3O3 (0.27 μM oligomer). *F*, quantified Rca activities of AfQ3O3 using inhibited carboxysomal *A. ferrooxidans* rubisco. *G* and *H*, activation time courses of *Halothiobacillus neapolitanus* rubisco in the presence and absence of HnQO using the same conditions as in *D* and *E*. *I*, activation of *H. neapolitanus* rubisco by different CbbQO activases at 5 and 13% v/v PEG (20 mM NaHCO_3_). Error bars indicate mean and standard deviation of three technical replicates. CABP, carboxy-arabinitol 1,5-bisphosphate; ECM, Enzyme-CO_2_-Mg^2+^; ECMC, ECM-CABP; Rca, rubisco activase.

Sutter *et al.* (30) demonstrated that the CbbQ protein encoded by the *H. neapolitanus* carboxysomal gene cluster physically associated with the α-carboxysome. Consistent with the requirement for association of the adaptor protein CbbO (26), in that study, the CbbQ hexamer was not able to activate inhibited carboxysomal rubisco. To clarify the function of the CbbQ and CbbO isoforms encoded by carboxysome gene clusters, we produced in *E. coli* and purified the form IA rubisco-associated CbbQO complexes encoded by *A. ferrooxidans* (IA^Q^-AfQ1O1 and IA^C^-AfQ3O3) and *H. neapolitanus* IA^C^ (HnQO) (Fig. 3*A*). The ATPase activity of the purified AfQ3O3 complex was similar to that of the AfQ1O1 complex. HnQO was much faster, showing an ATPase activity of ∼8 min^−1^/CbbQ protomer (Fig. 3*B*).

We then assayed carboxysomal AfQ3O3 for the ability to remove the transition state analog CABP from the *A. ferrooxidans* form IA^C^ (AfL2S2) holoenzyme (ECM), using conditions previously established for the *A. ferrooxidans* form IA^Q^ enzyme (26) (Fig. 3*C*). AfQ3O3 was almost nonfunctional, but increasing the concentration of the crowding agent PEG 3350 from 5% to 13% v/v resulted in an eightfold activity increase and thus robust Rca function (Fig. 3, *D* and *F*). The dependence of AfQ3O3 on high PEG concentrations was seen irrespective whether ECM component (ECMC) or ER (Fig. 3*E*) was used as inhibited rubisco, or whether the assays were performed in the presence of 5 or 20 mM bicarbonate (Fig. 3*F*). AfQ3O3 is therefore the activase of the carboxysomal AfL2S2 (form IA^C^) rubisco, which is encoded in the same operon (Fig. 1*A*).

HnQO was able to remove CABP from its cognate form IA^C^ rubisco HnLS even in the absence of PEG (Fig. 3*G*), although inclusion of the polymer also greatly enhanced Rca activity (Fig. 3, *G*–*I*). Titrating both HnQO and PEG concentrations in this assay demonstrated a concentration dependence for both components and showed that Rca function was similar at 0.8 μM HnQO/13% PEG and 3.2 μM HnQO/5% PEG (concentration expressed as CbbQ protomer) (Fig. S4). This finding is consistent with the dominant volume exclusion contribution of macromolecular crowding. Here, the crowding agent occupies a substantial fraction of the volume, and this results in a smaller available volume for both rubisco and activase, leading to higher biochemical activities (62, 63, 64). The strong positive effect of molecular crowding presumably relates to the carboxysomal microenvironment of AfL2S2 and HnLS. This property may be related to carboxysomal rubisco's coacervation with the multivalent rubisco linker protein CsoS2 (65, 66). A strong positive effect of PEG is also observed when assaying green-type plant Rcas, which are found in the crowded chloroplast stroma (67, 68).

The two Rca complexes from *A. ferrooxidans* (AfQ3O3 and AfQ1O1) were also able to remove CABP from HnLS but displayed much lower activities in combination with a great dependence on high PEG concentrations (Fig. 3*I*).

These data establish that the gene products of *cbbQ* and *cbbO* encoded by carboxysomal gene clusters in both *A. ferrooxidans* and *H. neapolitanus* are capable of reactivating inhibited carboxysomal form IA^C^ rubisco complexes. The function of the *A. ferrooxidans* activase was greatly enhanced by high concentrations of the molecular crowding agent PEG.

### The role of the RbcL C terminus in recognition by CbbQO

**Figure 4:**
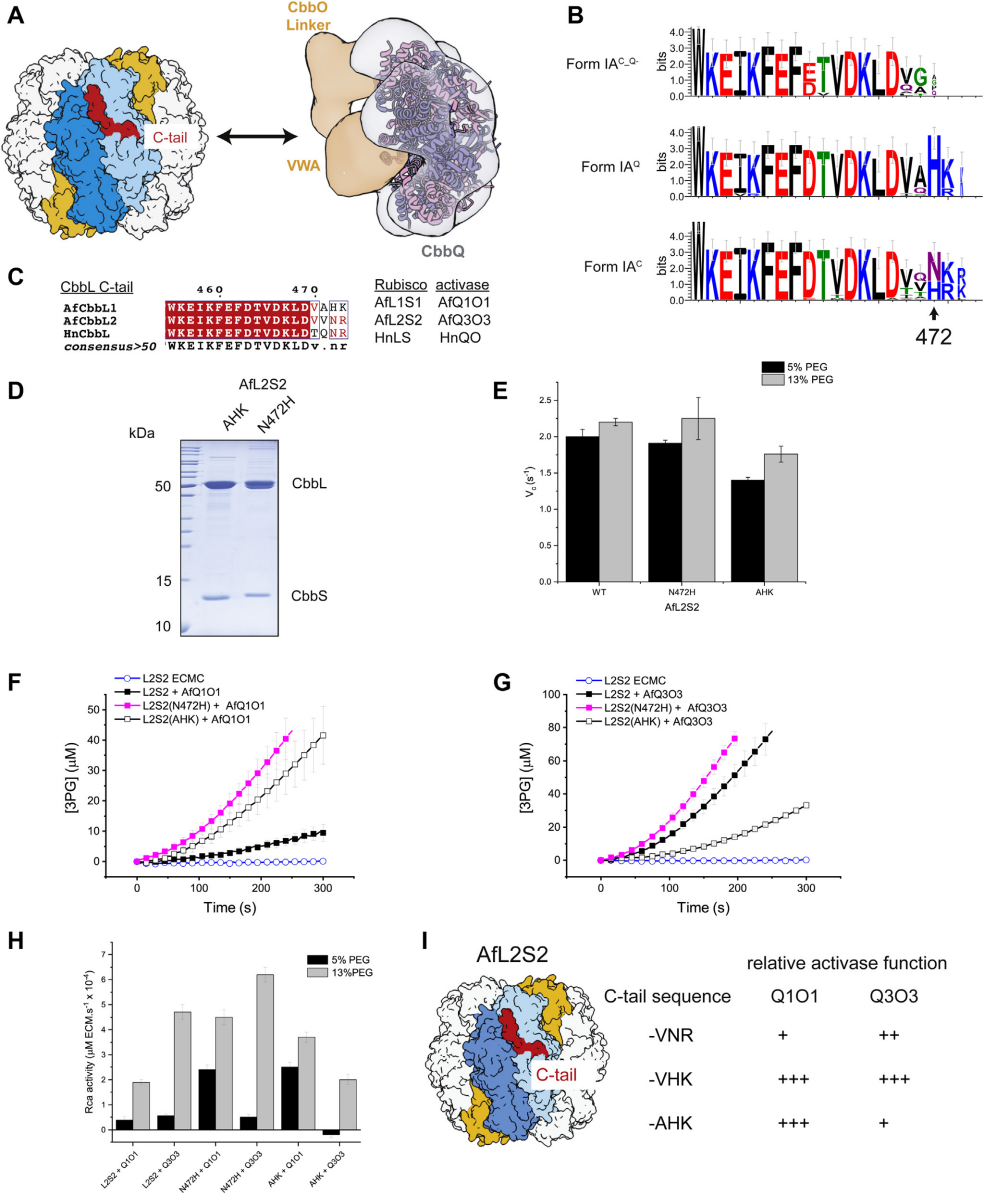
**The RbcL C terminus encodes Rca specificity.***A*, model of CbbQO function. The large subunit C terminus (*red*) is required for interaction with the activase and likely interacts with the CbbO adaptor protein. *B*, Logo motifs of the C termini generated from the RbcL sequences used for the phylogenetic tree in Fig. S1. *C*, alignment of the form IA RbcL C termini used in this study. *D*, SDS-PAGE analysis of purified AfL2S2 variants. AHK: the VNR C terminus is substituted with AHK. *E*, carboxylase activity of fully activated (ECM) AfL2S2 wildtype and variant proteins assayed at 20 mM NaHCO_3_. *F*, mutation at the C terminus of AfL2S2 enhances activation of ECMC complexes by Q1O1 (5% v/v PEG and 20 mM NaHCO_3_). *G*, the N472H mutation enhances AfQ3O3 function, whereas the AHK substitution reduces it (13% v/v PEG and 20 mM NaHCO_3_). *H*, quantified Rca activities for AfL2S2 and its variants. *I*, scheme summarizing how mutations of the RbcL C tail influence activase function. Rca, rubisco activase.

The RbcL C terminus is critical for CbbQO-mediated activation for both form I and form II rubiscos. The interaction is mediated by a C-terminal HK/R motif, and modification of the C terminus (including single-residue truncations and extensions) eliminates the ability of rubisco to become activated by CbbQO (26) (Fig. 4*A*). Carboxysomal rubisco large subunits that do not encode *cbbQ* and *cbbO* in the operon (FormIAC_Q^−^) are missing this motif and generally have a shorter C-terminal motif KLDXX. In contrast, when cbbQ and cbbO are present in the operon, the large subunit C-terminal motif is longer (KLDXXHK or KLDXXXR) (Figs. 4*B* and S2).

The form IA^C^ rubiscos from *A. ferrooxidans* and *H. neapolitanus* do not possess the conserved HK/R motif but instead encode an alternative “NR” C terminus (Fig. 4*C*). Two AfL2S2 rubisco C-terminal variants were produced and purified (Fig. 4*D*) and displayed similar carboxylation kinetics and spontaneous RuBP release under our assay conditions (Figs. 4*E* and S5). This permitted us to compare their activation kinetics using the two *A. ferrooxidans* CbbQO complexes AfQ1O1 and AfQ3O3.

Activation of the form IA^C^ AfL2S2 rubisco by either AfQ1O1 or AfQ3O3 was poor at 5% v/v PEG (Fig. 4, *F* and *H*). Substituting N472 with histidine or replacing the last three amino acids (VNR to AHK) resulted in a sixfold enhancement in AfQ1O1 activase function (Fig. 4, *F* and *H*). This indicates that the mutations significantly enhanced the low affinity of AfQ1O1 for the carboxysomal rubisco.

This dramatic increase in function was not observed when using the cognate AfQ3O3 assayed at 13% v/v PEG (Fig. 4*G*). Whereas AfL2S2N472H permitted a 30% increase in AfQ3O3 function compared with the wildtype AfL2S2, AfL2S2 (AHK) showed a ∼60% reduction in activation rate (Fig. 4, *G* and *H*). Substituting the last three residues of the C terminus therefore resulted in a specificity switch regarding the preference of the two *A. ferrooxidans* CbbQO complexes (Fig. 4*I*).

These results suggest that the as yet unidentified binding sites of *A. ferrooxidans* Q1O1 and Q3O3 to the RbcL C terminus are nonequivalent and can enable the activases to discriminate between substrate rubiscos. However, for both activases, a histidine residue at the penultimate position of RbcL is preferred to achieve high activase functionality *in vitro*. A crowded environment like the one found in a carboxysome may relax the requirement for a high rubisco–Rca binding affinity.

## Discussion

In this contribution, we conclusively demonstrate that CbbQO complexes encoded in proteobacterial carboxysomal operons are functional at removing inhibitory sugar phosphates from carboxysomal form IA^C^ rubisco active sites. The widespread evolutionary conservation of these genes, in conjunction with the functional green-type activase from *Nostoc* (40), therefore suggests that robust carboxysome CCM function relies on the ability to remove RuBP or other sugar phosphates from the rubisco active site in some bacteria. Our work does not inform on the nature of the physiological inhibitor(s), but these are likely to be reaction misfire products, such as xylulose 1,5-bisphosphate or possibly pentadiulose-1,5 bisphosphate (12, 13). Elucidation of this issue will necessitate a detailed biochemical study of rubiscos isolated from bacterial mutants with lesions in the associated *cbbq*/*cbbO* genes. RuBP is unlikely to be a relevant inhibitor because the concentration of Mg^2+^ and CO_2_ is expected to remain high in the carboxysome, which will disfavor decarbamylation and ER formation.

*A. ferrooxidans* possesses two form IA rubiscos, one form II enzyme and three CbbQO complexes (26, 56). Its form IA^C^ rubisco (AfL2S2) presents with a similar $k_{\text{cat}}^{\text{c}}$ and *K*_*m*_ (CO_2_) but a reduced specificity factor compared with the form IA^Q^ enzyme (AfL1S1) (Table 1). The tendency of AfL2S2 to spontaneously release RuBP from the apoenzyme is less than that of the other carboxysomal rubiscos tested but more rapid than the form IA^Q^ rubisco AfL1S1 (Fig. 2). Our work thus shows that three carboxysomal rubiscos possess highly variable carboxylase kinetics and inhibitor binding. A well-characterized parallel regarding kinetics is the situation in pyrenoid-containing diatoms, where a wide variety of kinetic constants are also observed and interpreted to relate to relative CCM strength (69). A stronger CCM will permit the selection pressure on *K*_*m*_ (CO_2_) and S_c/o_ to relax, and this will permit higher carboxylase velocities (57, 70). These properties may be related to the relative availability of metabolic energy and inorganic carbon for these organisms, which will determine the CO_2_ concentrations that can be achieved at the carboxysomal rubisco active site. It is possible that *H. neapolitanus* and cyanobacterial rubiscos experience higher CO_2_ concentrations because their metabolic modes (sulfur oxidation and oxygenic photosynthesis) generate copious amounts of energy. In contrast, *A. ferrooxidans* grows at low pH, which translates to an absence of the bicarbonate pool, and uses the energetically challenging iron-oxidizing metabolism (25). Its carboxysomal rubisco may thus be exposed to lower CO_2_ levels.

Our findings also show that in *A. ferrooxidans*, the CbbQO complexes are specific for the form IA rubisco encoded in their gene cluster, and that this specificity is dependent on the identity of the RbcL C terminus, which has been implicated in CbbQO function previously (26) (Fig. 4). This biochemical finding implies that in *A. ferrooxidans*, it will be important to package the correct (AfL2S2) rubisco in the carboxysome together with its AfQ3O3 activase. Recent discoveries elucidating the mechanisms of α-carboxysomal rubisco compartmentalization *via* the CsoS2 rubisco linker protein provide a straightforward experimental framework to explore these issues (65, 71).

The requirement for activase-mediated rubisco remodeling in carboxysome function has been controversial (30, 35), and in particular, the presence of *cbbQ* and *cbbO* in carboxysomal gene clusters has remained unexplained (45, 72).

In light of our findings, we propose that dependence of organisms bearing carboxysome CCMs on activase function will vary and relate to both phylogenetic history and to a continuum of kinetic and inhibitory properties of the associated carboxylases. These properties are sculpted by the prevailing CO_2_ concentrations experienced by the active sites during evolution (59, 69). On one extreme of the spectrum, there are carboxysomal rubiscos, such as the *A. ferrooxidans* form IA^C^ enzyme (AfL2S2), which do not readily release RuBP from the apoenzyme, and are only modestly faster than the cytoplasmic form IA^Q^ enzymes (AfL1S1). Such carboxysomal enzymes will be predicted to strictly coexist with Rca-encoding genes. At the other extreme of the continuum, the majority of cyanobacteria do not encode Rca homologs. Consistently, the fast Synechococcus form IB enzyme rapidly dissociates from inhibitory RuBP and XuBP (32). However, its *K*_*m*_ (CO_2_) is exceptionally high, and concordantly, the carboxylase efficiency $k_{\text{cat}}^{\text{C}}/K_{\text{C}}$ is poor.

The enzyme from *H. neapolitanus* is intermediate to the extreme examples. Inhibitory compounds are released more readily, the enzyme is fast and possesses a high *K*_*m*_ (CO_2_) but not as exaggerated as the cyanobacterial rubisco. In addition, experimental results suggest that deleting CbbQ or CbbO has a negligible effect on fitness under laboratory conditions (47). It is possible that a fitness defect would become apparent if growth under energy-limited conditions was explored (73). Transferring the *H. neapolitanus* carboxysomal genes and other CCM components into *E. coli* was successful, even if CbbQ was nonfunctional (46). It is possible that in this scenario, the fitness defect is too subtle to be detected in the context tested in the laboratory.

Although the outlined physiological data suggest that the *H. neapolitanus* CbbQO activase is not critical, here, we show that biochemically the system is functional. In addition, in our bioinformatics analysis of CbbL sequences, we did not encounter IAC_Q^−^ branches inside the form IA^C^/A^Q^ cluster (Fig. S1). Evolutionary loss of the CbbQO activase system thus appears to be a rare event. Finally, an article published during revision of this article compared *E. coli*-produced *H. neapolitanus* carboxysomes. It was found that if HnQO was incorporated, the carboxysomes were more functional at fixing CO_2_ in the presence of substoichiometric amounts of CABP (74).

Further systematic characterization of carboxysomal rubisco kinetics, their inhibitory properties, and activase dependencies will contribute to our appreciation of the carboxylase intrinsic tradeoffs (57, 75, 76, 77). It is likely that biotechnological exploitation of carboxysomes in plants and other organisms will benefit from taking into account a comprehensive understanding of these factors.

## Experimental procedures

### Bioinformatics analysis

BLAST searches were performed using the AfCbbL1 sequence (UniProt: B7JA24) to query the Integrated Microbial Genome database (78) and the National Center for Biotechnology Information. Classification into form IA^Q^, IA^C^, and IAC_Q^−^ was performed manually by inspecting the gene neighborhood of the selected genes. Maximum likelihood phylogenetic trees were constructed using MegaX (www.megasoftware.net) (79) and visualized using iTOL (https://itol.embl.de) (80). Positions containing gaps or missing data were eliminated. The sequences used are provided in Dataset S1. Multiple sequence alignments were drawn using ESPript (https://espript.ibcp.fr) (81). Gene neighborhoods were visualized using clinker (https://github.com/gamcil/clinker) (82).

### Molecular biology

Rubisco- and activase-encoding genes were amplified from genomic DNA of *H. neapolitanus* ATCC23641 and *A. ferrooxidans* ATCC23270. The *H. neapolitanus cbbL* (Hneap_0922) and *cbbS* (Hneap_0921) genes including the intergenic region were cloned between NdeI and HindIII sites of pET30b to yield pET30b*HnLS*. The carboxysome CbbQO-encoding genes *HncbbQ* (Hneap_0905)/*AfcbbQ3* (AFE_1678) and *HncbbO* (Hneap_0910)/*AfcbbO3* (AFE_1677) were cloned into pHue and pBad33 in the same context as pHue*AfCbbQ1* and pBad33*UbAfcbbO1* (26).

Site-directed mutagenesis was used to introduce mutations into the *AfcbbL2* C terminus. The corresponding primers and plasmids used in this study are listed in Tables S1 and S2. Protein-encoding sequences were verified using DNA sequencing.

### Proteins

Proteobacterial rubiscos were produced in *E. coli* BL21(DE3) cells transformed with the appropriate plasmid and purified using anion exchange chromatography followed by size-exclusion chromatography as described for AfL1S1 (26). The SynLS purification protocol was described (55).

Rca complexes were produced in *E. coli* BL21(DE3) harboring the appropriate plasmids (Table S2) and purified using a combination of affinity chromatography, ion exchange chromatography, and size-exclusion chromatography. The purification of AfQ1O1 was described previously (26). AfQ3O3 and HnQO were obtained using the same strategy except that the ion exchange chromatography step was omitted for AfQ3O3. Protein concentrations were quantified using the Bradford assay using bovine serum albumin as the standard.

### Enzymatic assays

All assays were performed at 25 °C. ATPase activity was measured using a coupled spectrophotometric assay (83). The CbbQO concentration used was 0.27 μM oligomer. RuBP was synthesized from ribose 5-phosphate and purified by anion exchange chromatography (84, 85). Rubisco and Rca assays were performed using the coupled spectrophotometric rubisco assay (26, 86) and quantified as described (87). ECM holoenzyme was obtained by incubating rubisco (10 μM active sites) in 20 mM Tris–HCl, pH 8.0, 50 mM NaCl, 50 mM NaHCO_3_, and 10 mM MgCl_2_ for 10 min at 25 °C. A fourfold molar excess of carboxypentitol bisphosphate (synthesized from RuBP following Ref. (88)) was added to ECM to form ECMC. Excess ligand was then removed by buffer exchange into 20 mM Tris–HCl, pH 8.0, and 50 mM NaCl using Micro-Bio-Spin 6 (BioRad) chromatography columns.

Inhibited ER complex (10 μM) was obtained by incubating apo-rubisco in 20 mM Tris–HCl (pH 8.0), 50 mM NaCl, and 4 mM EDTA for 10 min, followed by addition of RuBP to a final concentration of 0.4 mM for 30 min at 25 °C. Rca assays contained either 5 or 13% v/v PEG 3350 (Sigma–Aldrich).

Rubisco kinetics were determined using radiometric rubisco assays (89). ^14^CO_2_ fixation assays (0.5 ml of reaction volume) were performed at 25 °C in 7.7 ml septum-capped glass scintillation vials with assay buffer (100 mM *N*-(2-hyroxyethyl)piperazine-*N*'-(3-propanesulfonic acid)–NaOH, pH 8.0, 20 mM MgCl_2_, and 1 mM EDTA), 10 mg/ml carbonic anhydrase, and 1 mm RuBP. All the assay components were equilibrated with nitrogen gas prior to addition of ^14^CO_2_ varying from 2 to 40 mM NaH^14^CO_3_ (corresponding to 30–500 μM ^14^CO_2_). Purified rubisco (∼5 μM active sites) was first activated using assay buffer containing 30 mM NaHCO_3_, and the assay was then initiated by addition of 20 μl of activated rubisco. The assay was stopped after 2 min using 200 μl of 50% (v/v) formic acid. The specific activity of ^14^CO_2_ was measured using the highest ^14^CO_2_ concentration and 5.2 nmol of RuBP. The reaction was stopped after 30 min, and the activity was ∼220 CPM/nmol RuBP. Active site concentrations were determined using ^14^C-CABP binding assays (90). CO_2_/O_2_ specificity assays were performed as described (89, 91). The reactions were equilibrated in a defined gas mixture (999,300 ppm O_2_; 700 ppm CO_2_; Air Liquide Singapore Private Limited) prior to the addition of [1-^3^H]-RuBP.

## Data availability

All data are contained within the article and supporting information.

## Supporting information

This article contains supporting information (26, 55, 92).

## Conflict of interest

The Mueller-Cajar laboratory receives funding from Flagship Labs 79, Inc. All the other authors declare that they have no conflicts of interest with the contents of this article.
