# Supplementary material for: The CbbQO-type rubisco activases encoded in carboxysome gene clusters can activate carboxysomal form IA rubiscos
Source: J Biol Chem. 2021 Dec 8;298(1):101476. doi: 10.1016/j.jbc.2021.101476 (PMC8718961; doi:10.1016/j.jbc.2021.101476)
Supplement: Supplemental Figures S1–S5 and Table S1, S2 [file mmc1.docx]

**Supporting Information (SI)**

**The CbbQO-type Rubisco activases encoded in carboxysome gene clusters can activate carboxysomal Form IA Rubiscos**

Yi-Chin Candace Tsai^1^, Lynette Liew^1^, Zhijun Guo, Di Liu and Oliver Mueller-Cajar*

School of Biological Sciences, Nanyang Technological University, 60 Nanyang Drive,

Singapore 637551. Singapore

*Corresponding author: [cajar@ntu.edu.sg](mailto:cajar@ntu.edu.sg)

^1^ Y.-C.C.T. and L.L. contributed equally to this work


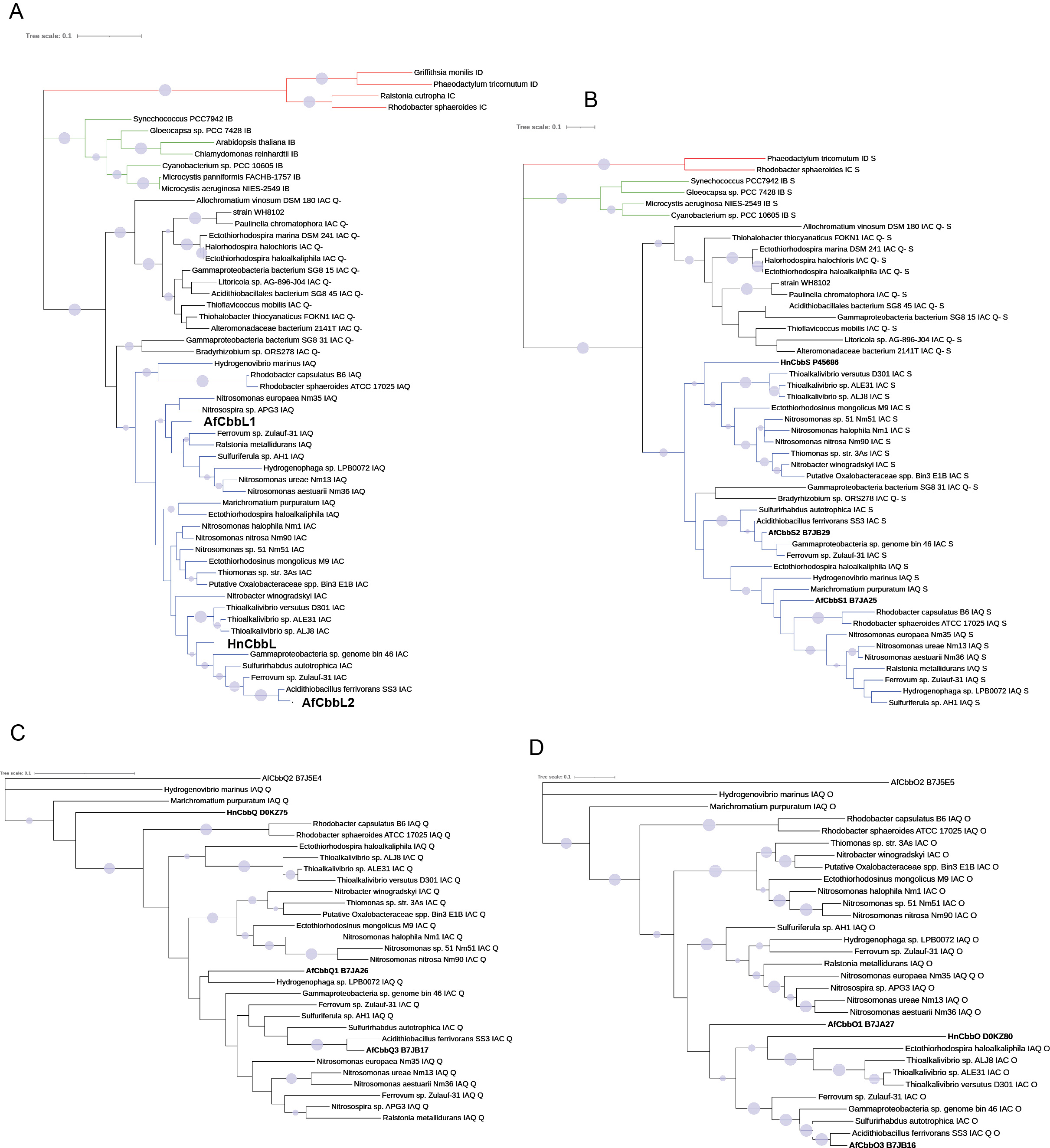


**Figure S1.** Maximum likelihood trees of Form IA Rubisco associated CbbL (A), CbbS (B), CbbQ (C) and CbbO (D) sequences. Sequences of the proteins used in this study are indicated by bold type. Bootstrap values are from 500 resamplings of the alignment and are indicated as circles when over 50%, with larger radii indicating stronger support. Trees were rooted either using red Rubisco sequences (A,B) or the Form II associated branches (C,D). IACQ- sequences are associated with a carboxysome gene neighbourhood lacking *cbbQ* and *cbbO*. IA^Q^ sequences are followed by *cbbQ* and *cbbO* and are not associated with carboxysome genes. IA^C^ sequences are followed by carboxysome genes and contain *cbbQ* and *cbbO* in the cluster.


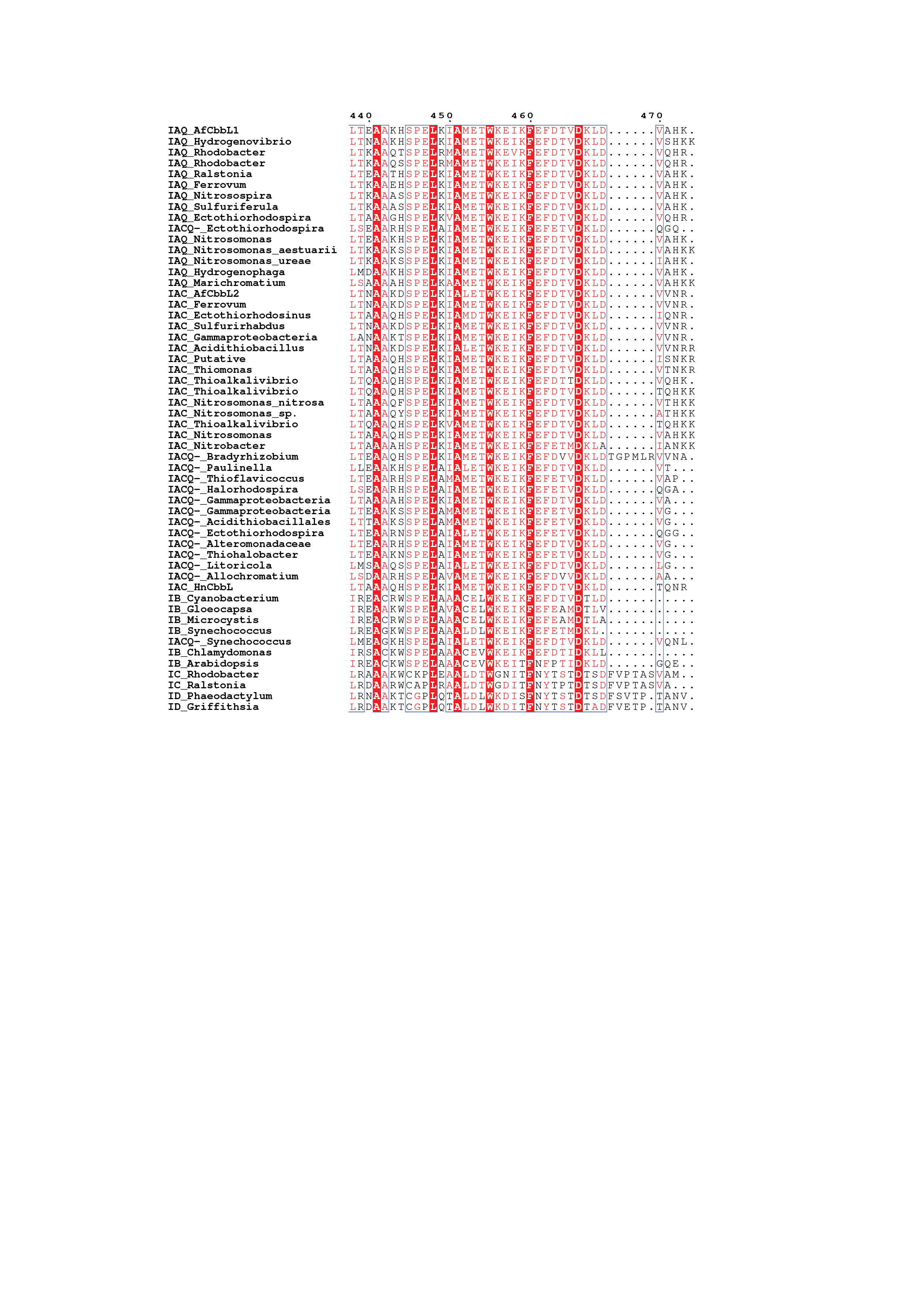


**Figure S2.** RbcL C-terminal sequences. Partial multiple sequence alignment of the Form I RbcL sequences used to generate the phylogenetic tree shown in Fig. S1. The presence of cbbQ and cbbO genes for IA^Q^ and IA^C^ sequences strictly correlates with the basic C-terminal CbbQO interaction motifs.


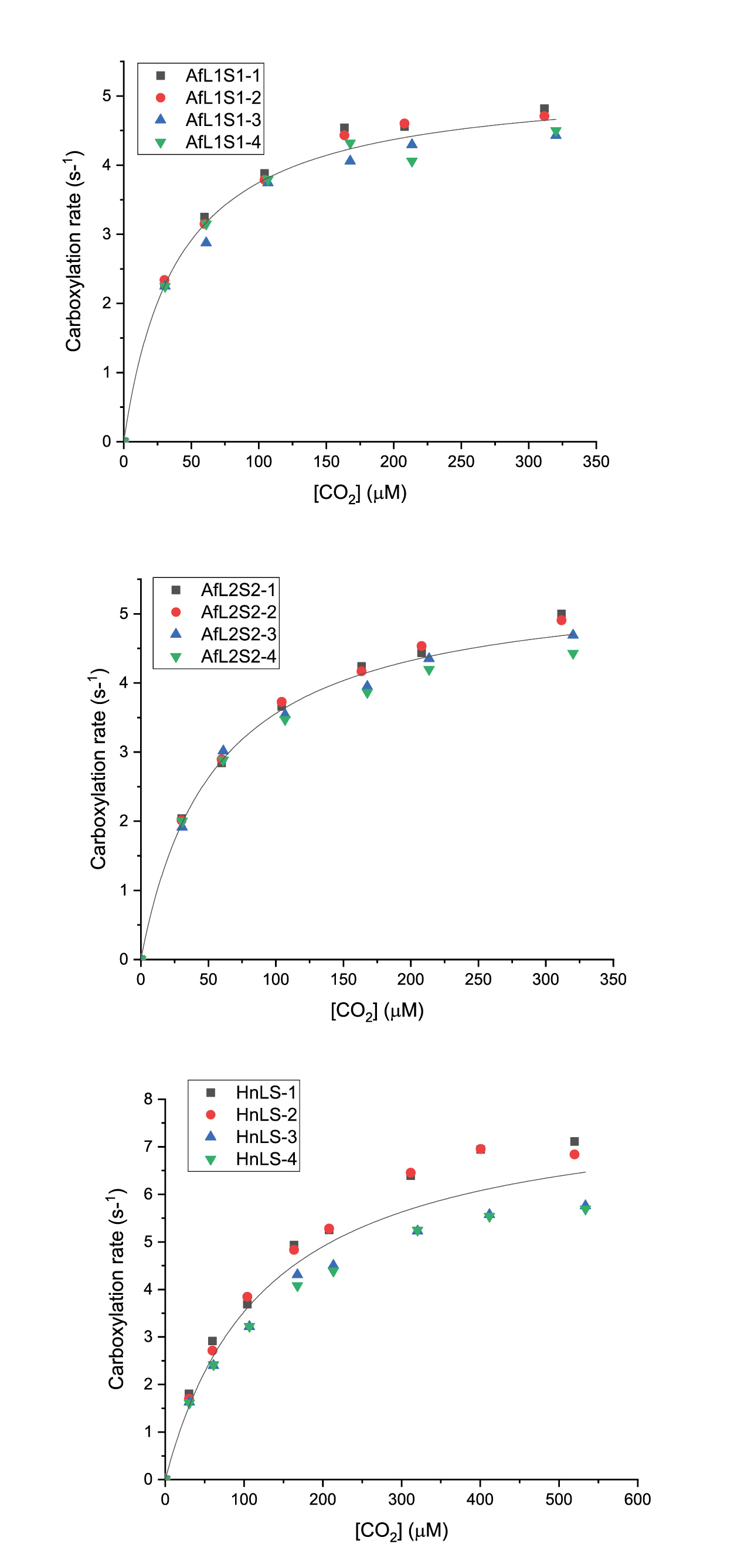


**Figure S3.** Kinetic characterization of Form IA Rubiscos. Data was collected in four technical replicates and the Michaelis-Menten model was fit to the concatenated data sets using Origin.


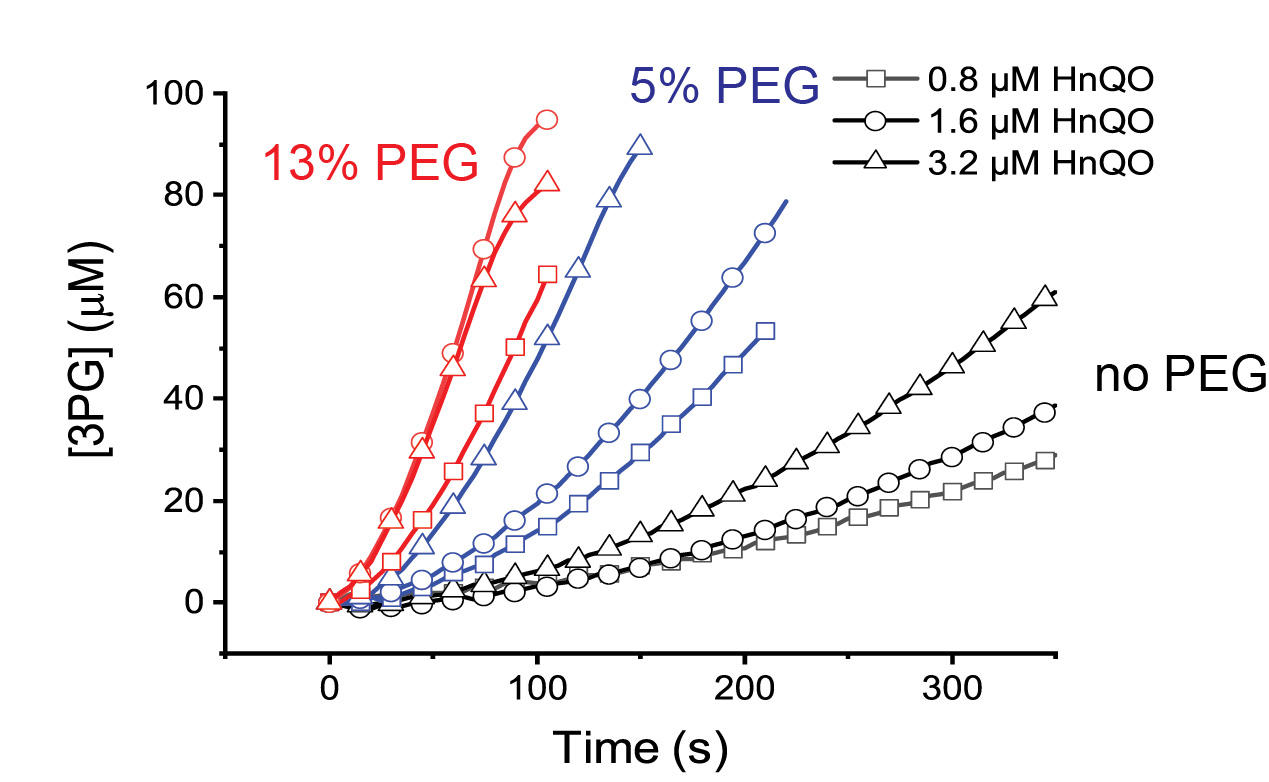


**Figure S4.** Titration of activase and PEG for the *H. neapolitanus* Rubisco-activase system. Rubisco activase assays contained 0.3 µM HnLS ECMC and the indicated concentrations of HnQO (expressed as protomer CbbQ, divide by six to obtain oligomer CbbQO) and PEG. Substrate concentrations used were 20 mM NaHCO_3_ and 1 mM RuBP.


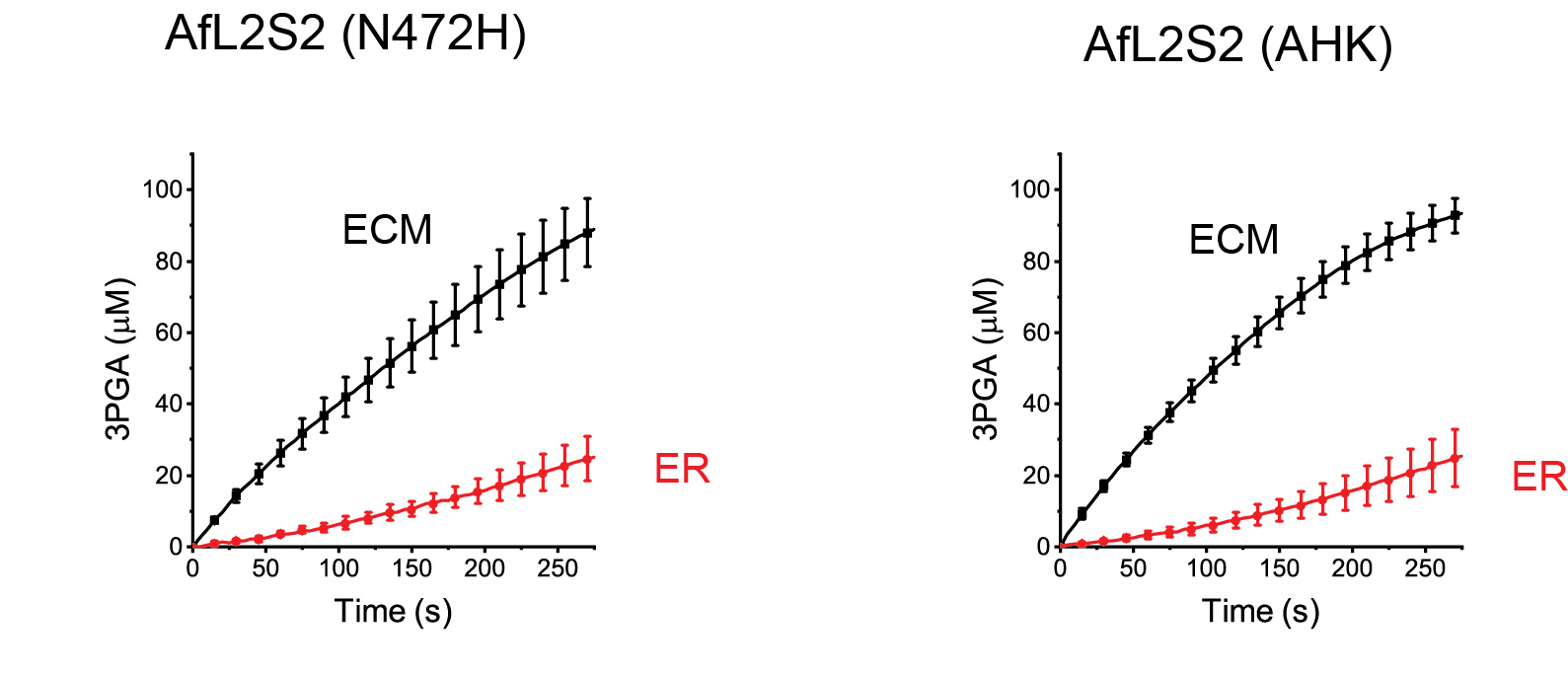


**Figure S5.** RuBP release kinetics of AfL2S2 mutants. Carboxylation time courses of 0.1 μM of fully activated holoenzyme (ECM) or apo-Rubisco-RuBP (ER) complex are shown (50 mM NaHCO_3_, 1 mM RuBP). Error bars signify mean and standard deviation of three technical replicates.

**Table S1. Primers used in this study**

| **Primer** | **Sequence (5’ to 3’)** |
| --- | --- |
| 5NdeIHnCbbL | AACATATGGCAGTTAAAAAGTATAGTG |
| 3HindIIIHnCbbS | TTAAGCTTTAGTTGCCGCGGTAGACCA |
| HnCbbLS(-HindIII)for | CTATGCGCAAAGTTTAGGTCTGGCC |
| HnCbbLS(-HindIII)rev | GGCCAGACCTAAACTTTGCGCATAG |
| 5SacIIHnCbbQ | AACCGCGGTGGTATGACACAAAATGCAGATCAA |
| 3HindIIIHnCbbQ | TTAAGCTTTAAAAGAACGTTTTGACGA |
| 5SacIIHnCbbO | AACCGCGG TGGT ATGAACCCAGCGACTGAAGAA |
| 3HindIIIHnCbbO | TTAAGCTTCTATCGCGTCATCGACAAATA |
| HnCbbO(-XbaI)for | TCAAGGCTTTTCTTGAAAGCGTTTCCA |
| HnCbbO(-XbaI)rev | TGGAAACGCTTTCAAGAAAAGCCTTGA |
| AfL2VNR471AHK_for | AATTGGACGTGGCACATAAATGATTCTGTGAC |
| AfL2VNR471AHK_rev | GTCACAGAATCATTTATGTGCCACGTCCAATT |
| AfL2N472H_for | TTGGACGTGGTGCATCGCTGATTCT |
| AfL2N472H_rev | AGAATCAGCGATGCACCACGTCCAA |
| 5AfQ3SacII | AACCGCGGTGGTATGTCACCAGAAATTGAT |
| 3AfQ3HindIII | TCAAGCTTCAGAAAAAAGTACTCACCGCC |
| 5AfO3SacII | AACCGCGGTGGTATGGCGGTCGAACTCGAA |
| 3AfO3HindIII | TCAAGCTTCATCGGGTGAGTCCCATGTAA |
| 5AfL2NdeI | GACATATGGCCGTAAAAAAGTATGAGG |
| 3AfS2EcoRI | TCGAATTCTTATCGCCCGCGGAACACCA |

**Table S2.** Plasmids used in this study

| **Plasmid(s)** | **Protein purified** | **Reference** |
| --- | --- | --- |
| pET30b*AfcbbLS* | AfL1S1 | Tsai et al. 2015 |
| pET24b*Af2cbbLS* | AfL2S2 | Wunder et al. 2018 |
| pET30b*HnLS* | HnLS | This work |
| pET24b*Af2cbbLSAHK* | AfL2S2AHK | This work |
| pET24b*Af2cbbLSN472H* | AfL2S2N472H | This work |
| pTrc*SynLS*,  pACYCH_6_*Ub^6301^S* | SynLS | Mueller-Cajar and Whitney (2008) |
| pHue*AfcbbQ1*, pBad33*UbAfcbbO1* | AfQ1O1 | Tsai et al. 2015 |
| pHue*AfcbbQ3*, pBad33*UbAfcbbO3* | AfQ3O3 | This work |
| pHue*HncbbQ*, pBad33*UbHncbbO* | HnQO | This work |
